# Taller staff occupationally exposed to less radiation to the temple in cardiac procedures, but risk higher doses during vascular cases

**DOI:** 10.1038/s41598-020-73101-4

**Published:** 2020-09-30

**Authors:** Kelly S. Wilson-Stewart, Davide Fontanarosa, Dan Li, Chris C. Drovandi, Rebecca K. Anderson, Jamie V. Trapp

**Affiliations:** 1 Cardiovascular Suites, Greenslopes Private Hospital, Newdegate Street, Greenslopes, Brisbane, QLD 4120 Australia; 2 Queensland University of Technology, 2 George Street, Brisbane, QLD 4000 Australia; 3 Institute of Health and Biomedical Innovation, Queensland University of Technology, Q Block, 60 Musk Avenue, Kelvin Grove, QLD 4059 Australia; 4 School of Mathematical Sciences, Queensland University of Technology, 2 George Street, Brisbane, QLD 4000 Australia; 5 Centre for Data Science, Queensland University of Technology, 2 George Street, Brisbane, QLD 4000 Australia; 6 ARC Centre of Excellence for Mathematical and Statistical Frontiers, The University of Melbourne, Parkville, VIC 3010 Australia

**Keywords:** Health occupations, Cardiology, Interventional cardiology

## Abstract

This study aimed to evaluate the effect of nurse and doctor height on occupational dose to the temple during fluoroscopically guided cardiovascular procedures. Additionally, an evaluation of the relationship between doctor height and table height was performed. Staff exposed during fluoroscopic procedures may be at elevated risk of cardiovascular damage or oncogenesis and have demonstrated a higher incidence of subscapular cataracts. The heads of taller staff may be exposed to reduced levels of radiation due to the increased distance from the area of highest intensity X-ray scatter. Limited research has been performed investigating height as a predictor of head dose to nursing staff. The level of radiation dose at the level of the temple to the doctor (n = 25), scrub (n = 28), and scout nurse (n = 29) was measured in a prospective single-center, observational study using Philips DoseAware badges. Procedural characteristics were recorded for vascular and cardiac cases performed in three dedicated angiography suites. Data were also collected to investigate relationships between doctor height and table height. Data were collected for 1585 cardiac and 294 vascular procedures. Staff height was a statistically significant predictor of temple dose for doctors, scrub, and scout nurses when considering the full data sample. The log temple dose demonstrated an inverse relationship to staff height during cardiac procedures, but a positive relationship for scrub and scout nurses during vascular studies. This observational study has demonstrated that taller staff are exposed to less cranial exposure dose during fluoroscopically guided cardiac examinations but has revealed a positive correlation between height and temple dose during vascular procedures. It was also determined that doctor height was correlated with average procedural table height and that vascular access point influences the choice of table elevation.

## Introduction

Catheter-based procedures have transformed the way patients are diagnosed and treated in recent decades^1^. Diagnostic and interventional cases require the use of X-ray imaging to allow the doctor to visualize the anatomical location and appropriate deployment of interventional devices. As implantable products and imaging technology improve, there has been an increase in the complexity and number of procedures undertaken, which creates additional concerns regarding radiation dose to the patient and staff^1,2^.

Any exposure to ionizing radiation carries an inherent risk of adverse effects on tissue at a molecular level and may result in tissue reactions not only to the patient but exposed staff as well^2^. To maintain adherence to the ALARA (as low as reasonably achievable) principle, it is essential to understand the factors which contribute to occupational dose so that effective mitigation strategies can be implemented to minimize exposure^3^. This observational study investigates the effect of staff height on occupational dose to the temple during fluoroscopically guided cardiovascular procedures.

## Background

In the United States, cardiovascular procedures contribute approximately 45% of the total cumulative effective dose to patients from all medical sources, excluding radiotherapy^4^. Given the assumed benefit to the patient and correct application of dose optimization techniques, this exposure can be justified^2^. In-room staff are also inadvertently exposed, receive no medical benefit, and may subsequently experience detrimental health effects. Resultant tissue effects following exposure to high levels of ionizing radiation have been well documented, but the impact of protracted, low dose radiation is less well understood^5^. Studies of occupationally exposed staff during fluoroscopic procedures have reported cardiovascular damage^6^, early vascular aging^7^ and an increased risk of oncogenesis^8,9^. Staff exposure to low levels of radiation has also resulted in a significantly higher incidence of subscapular cataracts when compared to unexposed populations^10–12^.

The intensity of scattered radiation is highest on the X-ray beam entrance side of the patient. Consequently, the X-ray tube is positioned under the operating table in modern fluoroscopy suites to reduce dose to the head, torso and upper extremities of staff^13^. One underpinning principle of radiation physics is the inverse square rule, which states that the intensity of radiation will decrease proportionally according to the inverse square of the distance from the radiation source^14^. Although scattered radiation from patient procedures is not a point-source and does not strictly follow the inverse square law, this dictum has implications for doctors and other in-room staff^2,15^, and may lead to the presumption that the increased vertical distance between the area of highest scatter and a taller staff member’s head may result in decreased dose.

There is limited research that investigates the effect of doctor height on occupational radiation exposure. Among the existing studies, there is almost universal agreement that height has an inverse relationship to dose (Table 1). To date, there is a notable lack of literature explicitly investigating the relationship between the height of staff other than the doctors, such as scrub and scout nurses, and occupational exposure during fluoroscopic procedures.

**Table 1:** Methodology and conclusion summary of previous investigations of staff height as a predictor of occupational dose during fluoroscopic procedures.

Author	Clinical (C)/phantom (P)	Doctor	Other staff	Measured location	Specialty	Result
Albayati et al.^16^	C (n = 17)	✓ (n = 3)	Assistant doctor Radiologist (n = 2)	Head Thorax	Peripheral vascular/radiology	Inverse relationship between doctor height and head dose No relationship between doctor height and thorax dose No relationship between assistant doctor height and measured doses
Kuon et al.^17^	P	✓	✗	Thorax	Cardiology	Inverse relationship between doctor height and dose
Principi et al.^18^	C (n = 13) P	✓ (n = 6)	✗	Eye	Radiology /cardiology	Inverse relationship between doctor height and dose A reduction factor up to 2 is observed for a 10-cm-taller doctor^a^
Rigatelli et al.^19^	C (n = 2630) P	✓ (n = 4)	Nurses (n = 9) Technicians (n = 7)	Thorax	Peripheral vascular/cardiology	Inverse relationship between staff height and dose for both clinical and phantom measurements independent on procedure type Result was consistent with/without the use of a CMLS^a^
Sciahbasi et al.^20^	C (n = 2028)	✓ (n = 6)	✗	Thorax Head Left wrist	Cardiology	Inverse relationship between height and dose measured at thorax
Willard et al.^31^	P	✓	✗	Head	Urology	Shorter surgeons receive higher scattered radiation exposure to the brain and eyes during fluoroscopy

*CMLS* ceiling-mounted lead shield; *cm* centimetre; *n* number of procedures in clinical studies, or the number of individual staff included in the study; ✓, staff role included in the study sample; ✗, staff role not included in the study sample; *C*, clinical study, *P*, phantom study

^a^Noteworthy.

## Aim

This prospective observational study aimed to evaluate the effect of nurse and doctor height on occupational temple dose during fluoroscopically guided cardiovascular procedures. A secondary aim assessed the legitimacy of the assumption that taller doctors operate using higher patient table levels during angiographic procedures.

## Methodology

Radiation dose levels were measured in a prospective single-center, observational study to evaluate occupational radiation exposure for the staff performing the roles of scrub nurse (n = 28), scout nurse (n = 29), cardiologist (n = 22) and vascular surgeon (n = 3) between February 2017 and August 2019. Approval was granted by the Ramsay Health Care QLD Human Research Ethics Committee (Protocol number—16/67) and informed, written consent was obtained from staff participants. Written informed consent was also obtained to publish identifying images in an online open-access publication. The research was conducted in accordance with the National Health and Medical Research Council guidelines.

Procedural characteristics and occupational dose data were collected for cardiovascular procedures performed in three dedicated angiography theatres using Philips Allura Xper equipment (Philips Healthcare, Best, Netherlands). Two of the systems had Clarity dose reducing software installed, and one did not (Room 1). As Room 3 was equipped with a larger detector and dedicated vascular software applications, the majority of the non-cardiac procedures, subsequently referred to as ‘vascular’, were performed in this room.

Case characteristics such as staff category, room, procedure, contrast type and volume, nature of any intervention, and access point were recorded. Cumulative procedural values for radiological parameters including fluoroscopy time, number of cine acquisitions, and patient dose information such as Dose Area Product (DAP) and Air Kerma (AK) were retrieved from the generated procedural dose reports and also documented. All of these variables were incorporated for possible selection in the regression model so that more accurate statements about the variables of interest could be obtained after accounting for other potential variables. Procedures were categorized as cardiac or vascular, diagnostic or interventional, and allocated depending on patient body part imaged.

### Staff height and temple dose

Discrete measurements of occupational radiation exposure were prospectively collected for the doctor (cardiologist or vascular surgeon), the scrub and scout nurse using Dose Aware badges (Philips Healthcare, Best, Netherlands). DoseAware is an active personal dosimeter (APD) that features a real-time exposure monitoring system. Previous research has stated that DoseAware responds satisfactorily in realistic fields in interventional cardiology scattered radiation fields^21,22^. In addition to individual calibration certificates supplied by the manufacturer, DoseAware readings have been verified when compared to thermoluminescent dosimetry by the Institut de Tècniques Energètiques—Universitat Politècnica de Catalunya, Barcelona^21^. Individual badge calibration was also tested before the commencement of data collection.

DoseAware dosimeter badges have an operational quantity of Hp(10) and an X-ray dose rate range and linearity of ± 10% 40μSv/h–150 mSv/h (Sievert)^23^. Ideally, eye dose should be measured using Hp(3) dosimeters, unfortunately, at the time of data collection, commercially available dosimeters specifically calibrated to Hp(3) were uncommon. It has been noted that DoseAware worn close to the eye can provide appropriate dose estimates^21^, but the response may present an overestimation of 5–15%^22^.

Given the varying rotational position of the X-ray tube during procedures, the International Electrotechnical Commission recommends an angular response from 0^0^ to 60^0^ for the energy range 20–100 kev^24^. Philips states an angular response of ± 5% within ± 5°, ± 30% within ± 50° and + 200%/− 100% within ± 90° for an energy range up to 120 kev^23^.

When scrub nurse or doctor were located with their left side closest to the X-ray tube, as in a coronary angiogram, badges were worn near the left temple which has previously been demonstrated to be the preferred badge position for measuring eye dose^25^. For procedures where the staff worked on the opposite side of the patient, such as insertion of permanent pacemakers (PPM), badges were worn on the right temple. Staff wore the badge according to where they anticipated they would be located for the majority of the case. The most common position adopted by the scout nurse during procedures was nearest the doors to the room; generally 2–3 m behind the scrub nurse and consequently badges were typically situated on the left temple. Dosimeter badges were located on the left temple of staff in > 97% of procedures.

Coronary cases, including left ventriculograms (LV) and aortograms, were performed with a cine and fluoroscopy rate of 15 frames per second (fps). PPM and electrophysiology (EP) procedures had a standard low dose acquisition protocol of 7.5 fps for both cine and fluoroscopy. Endovascular cases used a fluoroscopic frame rate of 7.5 fps. Digital subtraction angiography (DSA) acquisitions varied from 6 fps for endovascular aneurysm repairs (EVAR), 3 fps for abdominal and pelvic imaging, and reduced to 0.5 fps for distal leg vessels.

**Figure 1:**
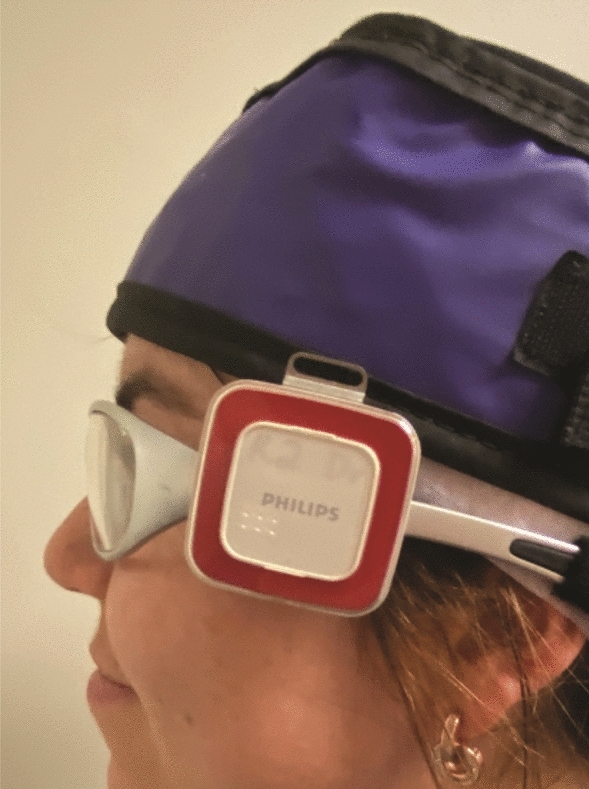
Position of DoseAware badge when worn attached to glasses.

**Figure 2:**
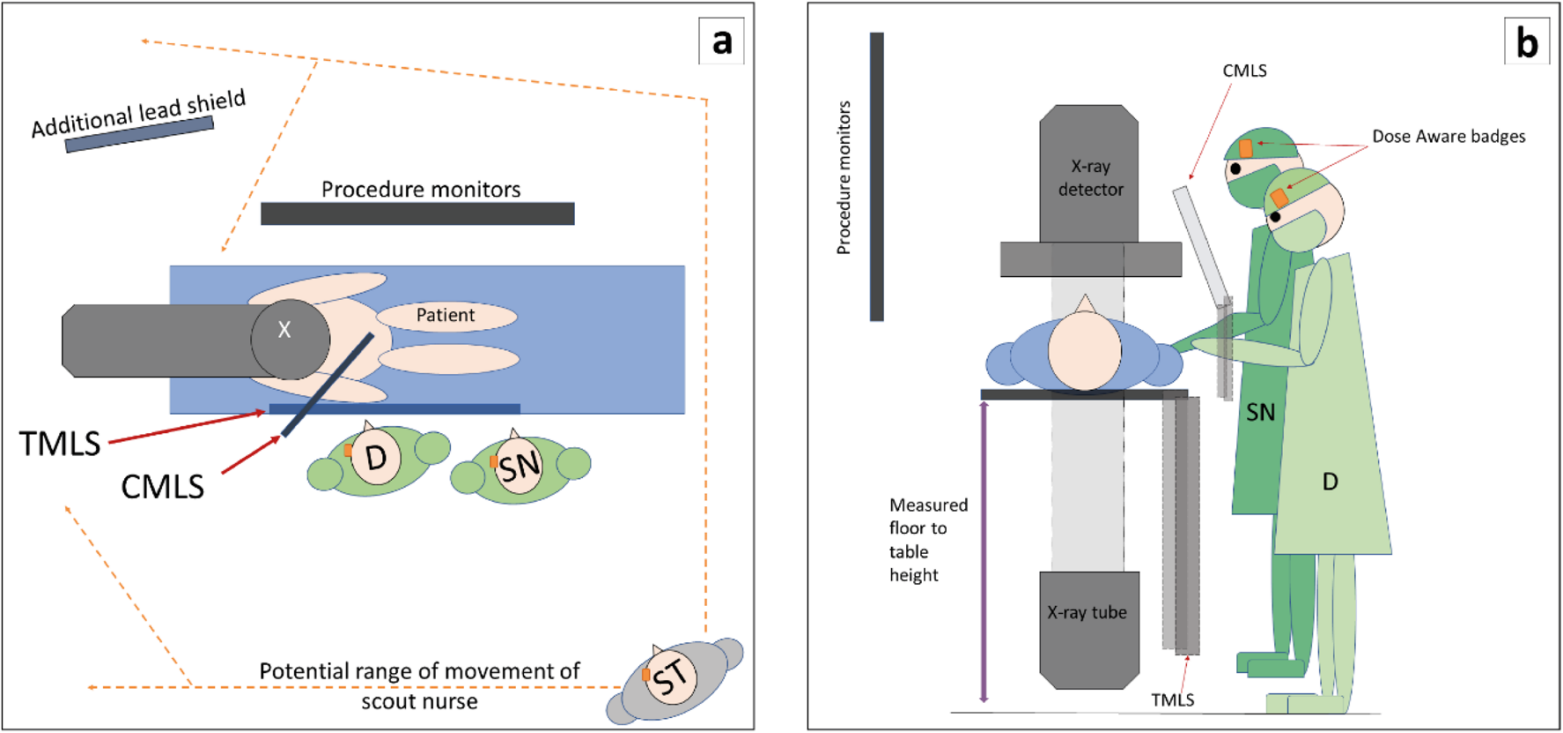
(**a**, **b**) Typical room setup and staff location. *CMLS* ceiling-mounted lead shield, *TMLS* table-mounted lead shield, *D* doctor, *SN* scrub nurse, *ST* scout nurse.

All doctors had a minimum of 15 years’ clinical experience in catheter-based cardiovascular procedures. All staff wore lead gowns and thyroid shields during cases, and most doctors and scrub nurses opted to wear lead glasses. Scrub and scout nurses routinely utilized leaded skull caps, while less than 10% of doctors chose to wear them. Depending on staff preference, the badge was either clipped external to the glasses, as demonstrated in Fig. 1 or fixed to the outside of the skull or theatre cap. Both ceiling-mounted lead shields (CMLS) and table-mounted lead shields (TMLS) were consistently used, and an extra movable lead shield was regularly utilized to provide additional protection to the scout nurse as shown in Fig. 2a. The relationship between the height of scrub nurse, scout nurse and doctor, and the levels of occupational temple dose was evaluated.

### The impact of doctor height on table height

To determine whether doctor height was correlated with average procedural table height (Fig. 2b), data were collected in 202 cases. The procedural average was determined by documenting table height data from the imaging system during individual fluoroscopic or cine events and calculating the mean table height for each procedure. Other potentially influential variables such as room number, access point and patient body mass index (BMI) were also recorded. At the author’s institution, the radiographer controls the X-ray tube geometry and the table height, and hence the effect of individual radiographers on table height was also investigated. Occupational dose was not examined.

### Statistical analysis

#### Staff height and temple dose

Linear regression models were applied to the data with the staff temple dose (log-transformed) as the response variable with the potential explanatory variables, such as staff role/height, patient BMI, room number, procedure type, imaged body part, entry point, and patient/case dose parameters. Two-way interactions between covariates were also considered. If a covariate appeared in a significant interaction, it was also included as a main effect regardless of whether the main effect was significant or not. The Akaike Information Criterion (AIC) was used to select among competing regression models^26^.

#### The impact of doctor height on table height

A linear regression analysis was performed to determine the potential effect of dr height, room number, radiographer, vascular access point and patient BMI on the procedural table height.

## Results

### Staff height and temple dose

Data were collected for 1585 cardiac and 294 vascular procedures. Males constituted approximately 70% of patients, and mean patient BMI was 29.6 for coronary and 28.4 for vascular procedures. Basic procedural information is presented in Table 2. Routine tube angles utilized for both coronary and vascular procedures are provided in Table 3. Staff height ranged from 155 to 190 cm for nurses and 167.5–192 cm for doctors, with the mean and quartiles represented in Table 4. The average dose per procedure was 3.14 μSv for the cardiologist and 6.6 μSv for the vascular surgeon. The scrub nurse received a mean dose of 2.8 μSv/5.6 μSv and the scout nurse a dose of 2.0/0.50 μSv for cardiac and vascular procedures, respectively.

**Table 2:** Procedure type and case numbers.

Procedure category	Procedure type	Number
**Coronary**	Coronary angiography—diagnostic	866
	Coronary angiography—interventional	534
Total		1400
**TAVI**	TAVI workups	21
	TAVI/Balloon valvuloplasty	24
Total		45
**Implantables**	PPM	67
	Watchman/amulet	14
	ASD/PFO closure	9
	Balloon pump	1
Total		91
**EP Total**	Diagnostic and interventional	48
**Vascular**	Abdominal angiography—diagnostic	20
	Abdominal angiography—interventional	42
	Abdominal + lower limb/s—diagnostic	28
	Abdominal + lower limb/s—interventional	48
	Lower limb diagnostic	25
	Lower limb interventional	68
	Subclavian/fistulogram/carotids—diagnostic	5
	Subclavian/fistulogram/carotids—interventional	20
	EVAR/FEVAR	38
Total		294

*TAVI* transcatheter aortic valve implantation, *PPM* permanent pacemaker, *ASD* atrial septal defect, *PFO* patent foramen ovale, *EP* electrophysiology, *EVAR* endovascular aneurysm repair, *FEVAR* fenestrated endovascular aneurysm repair.

**Table 3:** Routinely utilized procedural tube angles.

Procedure	LAO ↑ (35°/25°)	LAO ↓ (40°/35°)	RAO ↓ (20°/20°)	RAO ↑ (30°/30°)	AP ↑ (10°/35°)	AP (0°/0°)	RAO (30°)	LAO (30°)	LAO ↑ (25°/10°)	RAO (20°)	LAO (20°)	LAO (40°)
**Cardiac**												
Coronary angiogram												
LCA	√	√	√	√	√							
RCA							√	√	√			
LV							√					
TAVI workup	√	√	√	√	√	√	√	√	√			√
TAVI						√^a^				√	√	
PPM						√		√		√		
EP						√		√		√		
**Vascular**												
EVAR						√				√ (10° ↓)	√ (10° ↓)	
Abdominal aorta						√						
leg						√						
subclavian						√						
fistulograms						√						

*LCA* left coronary artery, *RCA* right coronary artery, *LV* left ventriculogram, *TAVI* transcatheter aortic valve implantation, *PPM* permanent pacemaker, *EP* electrophysiology, *EVAR* endovascular aneurysm repair, *LAO* left anterior oblique, *RAO* right anterior oblique, *AP* anterior–posterior, ↑ cranial angulation, ↓ caudal angulation.

^a^Individually customized implantation angle.

**Table 4:** Staff height per procedural category.

Procedure type	Staff role	N	Mean	Std Dev	Min	1st quartile	Median	3rd quartile	Max
**Coronary**	Doctor	1234	174	6.2	168	169	172	178	192
	Scrub	1147	173	10.0	157	165	170	181	190
	Scout	677	171	9.3	157	165	167	178	190
**Vascular**	Doctor	194	179	6.5	168	176	176	185	185
	Scrub	231	170	9.6	155	165	166	178	190
	Scout	143	173	9.9	160	166	174	180	190

Measured in centimeters (cm) expressed as mean, standard deviation, minimum, 1st Quartile, median, 3rd Quartile and maximum.

Staff height was a statistically significant predictor of temple dose for doctors, scrub and scout nurses when considering the full data sample. To quantify the impact of staff height on the temple dose with respect to a specific categorical variable that has significant interaction with staff height, other significantly interactive variables were considered. For different selected values of the interactive continuous variable, contrast volume, the impact was averaged over other interactive categorical variables.

**Figure 3:**
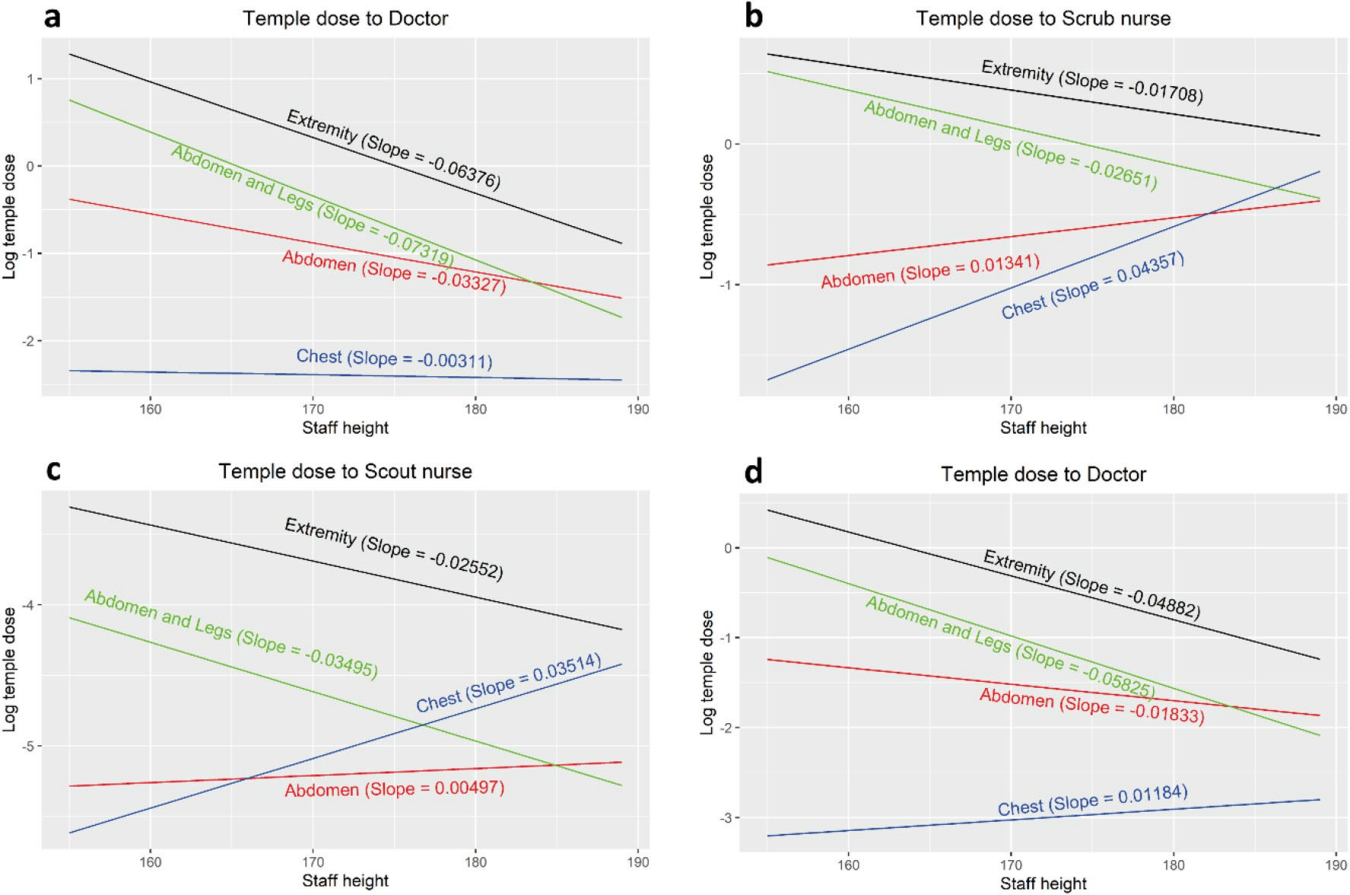
Impact of staff height on log temple dose with respect to body part imaged and different staff roles, doctor (**a**), scrub nurse (**b**), and scout nurse (**c**), for the median value of contrast volume. (**d**) demonstrates doctor dose with 95% quantile of contrast volume.

The imaged area of the patient’s body was found to influence temple dose with a decrease in exposure for taller staff (doctor, scrub and scout) within procedures performed on both the abdomen and extremities (predominantly aorto-femoral angiograms) and the extremities (Fig. 3a–c). For abdominal imaging, including endovascular aortic repairs (EVAR), and chest imaging, including transcatheter aortic valve implantation (TAVI) procedures, there was a positive relationship between staff height and temple dose for both scrub and scout nurses regardless of contrast volume. This relationship was also demonstrated for doctors in procedures within the chest (Fig. 3d) when contrast usage was 146 ml or 266 ml (75% and 95% quantile, respectively), but the impact of doctor height on temple dose was negative for procedures in which the contrast volume equalled 15 ml, 60 ml or 95 ml/60/95 ml (5%, 25% and 50% quantile, respectively).

**Figure 4:**
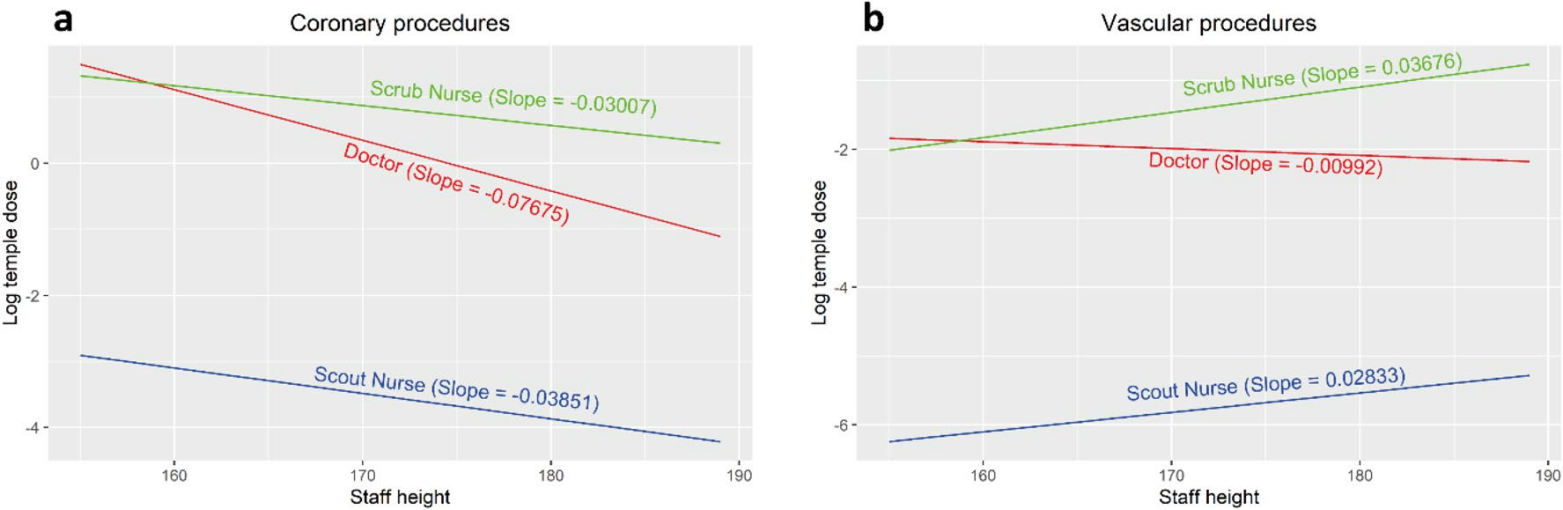
Impact of staff height on log temple for coronary (**a**) and vascular (**b**) procedures and different staff roles, for the median value of contrast volume.

Coronary and vascular procedures were separately analyzed, and the relationship between occupational temple dose and the doctor, scrub nurse and scout nurse height are presented in Fig. 4. As expected, taller staff are exposed to less cranial exposure during cardiac procedures, but surprisingly this trend was reversed for scrub and scout nurses during vascular cases.

Table 5 demonstrates the changes for log temple dose for different staff height during coronary and vascular procedures, again highlighting the trend of increasing temple dose to taller staff members during vascular procedures.

**Table 5:** Log temple dose change for different levels of staff height.

Procedure type	Contrast (ml)	Staff height (reference level: 160 cm)		
		170 cm	180 cm	190 cm
Coronary	15	− 0.55	− 1.11	− 1.66
	60	− 0.52	− 1.02	− 1.53
	95	− 0.48	− 0.97	− 1.45
	146	− 0.44	− 0.88	− 1.32
	266	− 0.34	− 0.67	− 1.01
Vascular	15	0.11	0.23	0.34
	60	0.15	0.31	0.46
	95	0.18	0.37	0.55
	146	0.23	0.46	0.69
	266	0.33	0.67	1.00

### The impact of doctor height on table height

Procedural table height was collected in all three rooms for 21 doctors, and for a total of 202 procedures (coronary n = 192; vascular n = 10) as shown in Table 6.

**Table 6:** Procedures, doctors and average table height.

Room	procedures	No. doctors	Mean TH	Std Dev TH	Min TH	Max TH
1	62	4	92.5 cm	19.9	86	110
2	88	16	91.6	2.2	86	99
3	52	16	91	2.09	85	95

Measured in centimeters (cm).

*TH* table height.

**Figure 5:**
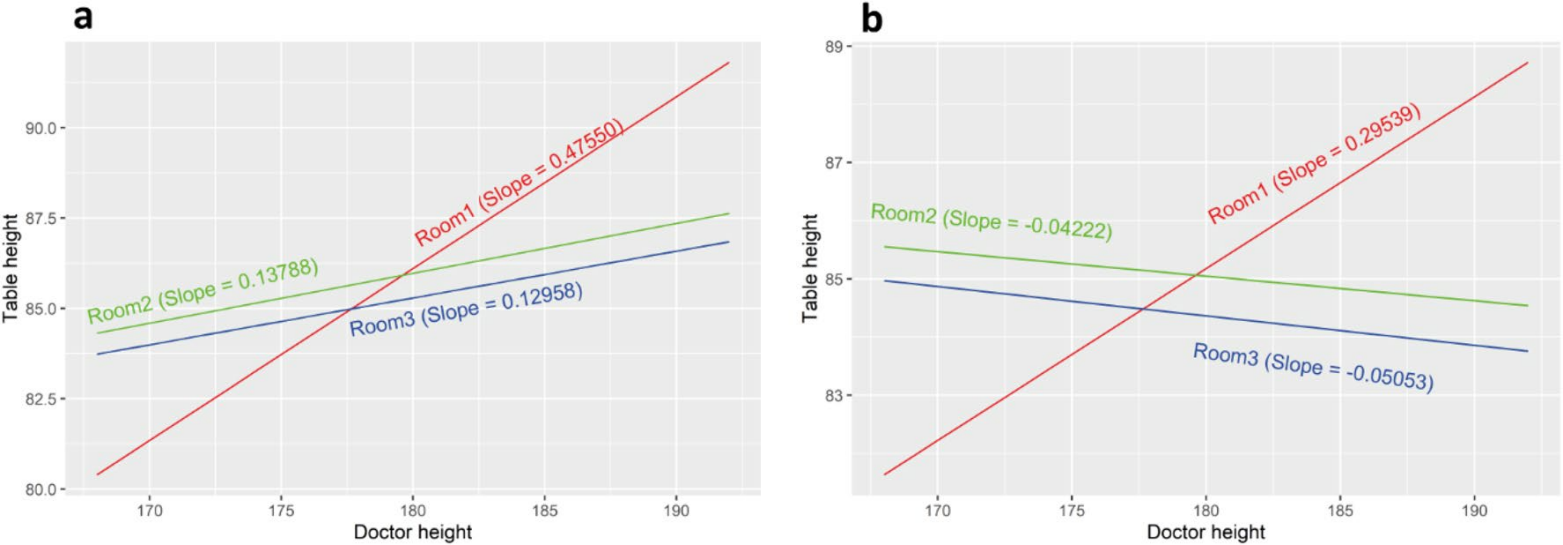
Impact of doctor height (cm) on table height for the median patient BMI (**a**) of 28.40 and the 95% quantile BMI (**b**) of 38.61.

There is strong evidence of a positive linear relationship between doctor height and procedural table height in room 1(Fig. 5a). The positive relationship in rooms 2 and 3 could become negative with a high patient BMI such as the 95% quantile BMI (Fig. 5b). There was little evidence of any impact on table height by individual radiographers.

**Figure 6:**
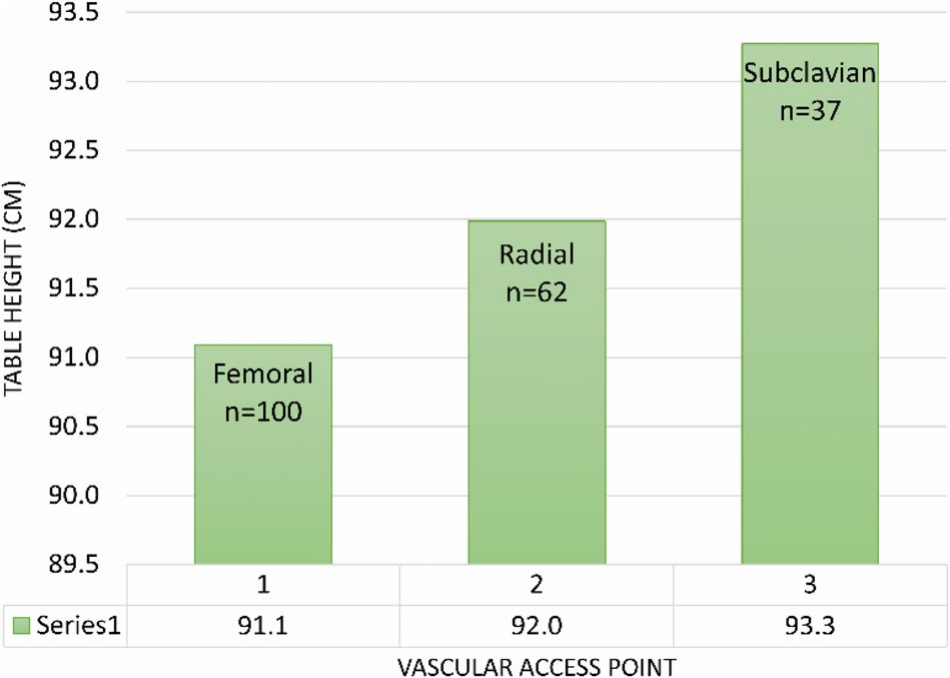
Average table height (cm) for different vascular access points.

The vascular access point had a statistically significant effect on procedural table height, and the average table heights for different vascular access points are demonstrated in Fig. 6.

## Discussion

### Staff height and temple dose

**Figure 7:**
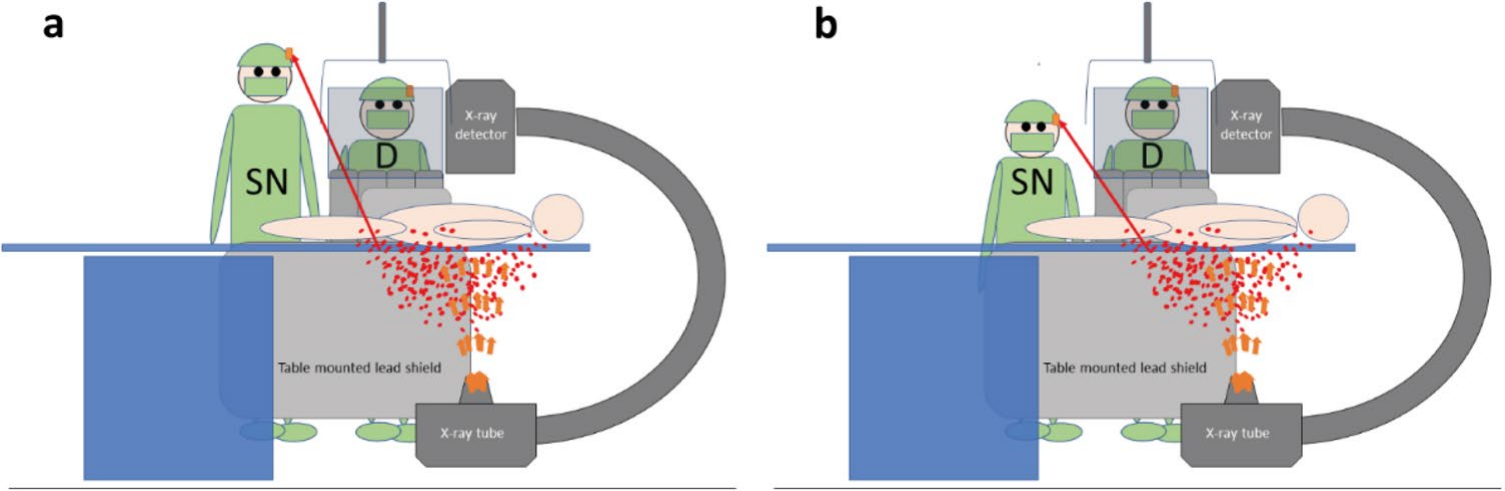
The heads of taller staff (**a**) may be exposed to less scattered radiation than shorter staff (**b**). *D* doctor, *SN* scrub nurse.

It stands to reason that the taller the staff member, the larger the distance between their head and the source of greatest scatter and consequently should be exposed to lower levels of cranial radiation than their shorter counterparts (Fig. 7).

There has been limited research investigating the effect of doctor height on occupational head dose, but of the available studies, the majority have demonstrated an inverse relationship between height and dose (Table 1). Kuon et al.^27^ found that scatter entrance skin dose decreased with greater body height, with taller doctors having a reduction in mean doctor head dose of approximately 1%/cm. Albayati et al. report a fivefold increase in measured dose for a shorter doctor (170 cm) when compared to taller colleagues, despite procedural parameters being similar^16^. Comparable to previous studies, analysis of the combined cardiac dataset reveals an overall inverse relationship between staff height and radiation dose to the head.

There is a notable paucity of literature explicitly evaluating the height of additional in-room personnel as a potential predictor of dose, and this may be due to the assumption that the proximity of the doctor to the primary source of X-ray scatter results in an additional dose. Rigatelli et al. measured dose in the clinical setting at the chest pocket level and classified doctors, nurses and technologists into two groups: those > 165 cm and those < 165 cm and reported significantly lower doses to taller individuals than their shorter colleagues, independent on procedure type, staff position or the use of a CMLS^19^. The effect of height on doses to individual staff roles in the clinical setting was not specifically articulated or discussed. Albayati el al. demonstrated no relationship between height and dose to an assistant doctor (radiologist)^16^. If it were assumed that the procedural positioning of an assistant doctor is similar to that of a scrub nurse, this investigation reveals important differences.

The results of this study indicate a strong positive height-temple dose relationship for scrub nurses after averaging over other categorical variables that have significant interactions with height for a collection of sample quantiles values of contrast volumes of 15 ml, 60 ml, 95 ml, 146 ml, and 266 ml (i.e. 5%, 25%, 50%, 75%, and 95% quantile, respectively), which contrasts with the previous (albeit limited) studies. BMI was found to affect dose levels to staff. This is unsurprising as the greater thickness of tissue an X-ray beam has to traverse, the higher number of X-rays are required, with a proportionally more substantial amount of scattered radiation incident on staff, which has been demonstrated previously^28^. When relatively large volumes of contrast were used, this was also shown to alter the trends of occupational dose as a predictor of temple dose, and this effect is understandable as larger volumes of contrast administered during an angiogram may be an indicator of greater procedural complexity.

Scrub nurses are generally positioned further away from the most significant source of x-ray scatter, which suppositionally reduces their level of exposure compared to the doctor. Still, it has been reported that nursing staff may be exposed to higher levels of radiation than the doctor due to the shelter provided by the CMLS^28–30^, and this may be a contributing factor in the increased dose to scrub nurses. The presence of the X-ray detector has also been identified as providing an incidental protective barrier to doctors’ heads by absorbing X-ray photons, depending on the position of the doctor^18,31^. While these elements may rationalize higher doses to the nurses, they do not explain the positive height-dose relationship for nurses, or why the effect is more pronounced for procedures which image the chest or abdomen.

One possibility is that the CMLS, while not providing the same level of protection afforded to the doctor, nevertheless offers a degree of cover from scattered radiation. In this case, the heads of shorter nurses would more likely be protected by this shield regardless of the doctor's height, and related positional height of the CMLS. In contrast, the heads of taller scrub nurses may extend above the lead glass, especially when the doctor may position it lower, allowing for scattered radiation to project above the shield and impinge on the cranium of the scrub nurse. It has been demonstrated that doses during endovascular procedures may be higher than that of coronary^32,33^ and this may explain why there is a marked difference between both the height-dose relationship when comparing coronary procedures to vascular (Fig. 4), but again fails to account for the positive height-dose relationship for scrub and scout nurses during procedures imaging the chest, which largely constitutes cardiac procedures.

It should be acknowledged that there is a wide range of scatter radiation at the level of the doctors’ eyes due to the variability of tube position^34^ and routine angles may differ between institutions or due to doctor preference. Procedures routinely utilizing steeper left anterior obliques (LAO) or LAO cranial tube angles provided in Table 3 may increase the risk of occupational exposure^17,20^. Additionally, these measurements were taken at a single point, and due to the changing nature of the X-ray scatter profile may not reflect dose levels to other areas of the head.

When extrapolated to anticipated yearly caseloads, the mean procedural doses for all staff categories during both vascular and cardiac procedures were < 2 mSv/year, which is well below the ICRP recommended yearly limits of 20 mSv/year^1^.

### The impact of doctor height on table height

Some authors have made the assumption that taller doctors would utilize a higher table position for ergonomic preference^20,35,36^, and this was corroborated by Faroux et al.^37^. Although this presumption seems logical, the interdependence was examined in the local clinical environment before this premise was applied to the findings of this study.

As expected, doctor height positively influenced table height when the patient’s BMI was not extremely high (Fig. 3a), with the strongest interrelationship demonstrated in room 1. The vascular access point was found to influence table height, with the highest average table height found to be during the insertion of pacemaker wires via the subclavian vein (Fig. 6). This association would be anticipated, as the shoulder is generally a much thinner body part than the hip, and the superficial location of the subclavian vein would lead itself to a higher table elevation. No relationship was demonstrated between individual radiographers and table height.

The positive relationship between doctor height and table height was mitigated with an increase in patient BMI; for instance, the previous positive relationship between table height and doctor height was negative when the patient’s BMI was at the 95% quantile level (BMI = 38.61). It is postulated that this may be due to larger patients having a skin access point higher above the surface of the table than that of a thinner patient, requiring a lower table height for doctor comfort.

Studies with similar case numbers^20^, as well as comparable procedures^19^, report an inverse relationship between height and dose measured at the level of the thorax, which is similar to the findings for the cardiac cases included in this investigation. In contrast to this, the results of this study indicate that nursing staff receive higher levels of occupational radiation dose to the left temple during procedures which image the chest and the abdomen and show a positive height-temple dose relationship during vascular procedures. This study investigated the dose to 28 nursing staff and 25 doctors, as opposed to Rigatelli et al.’s four doctors, nine nurses and seven technicians, and Sciahbasi et al.’s six doctors, which may also explain the discrepancy in results due to the increased number of variables. The measurement of dose at the height of the temple, as opposed to the thorax in this study, may have also contributed to the disparity in results.

## Limitations

This study was an observational design based on data from a single-center, which represents the most significant limitation. Specific procedural protocols, imaging equipment, and staff operational preferences are potentially limiting to the generalisability of the results.

Another limitation is the assessment of the effect of staff height on occupational doses at a single anatomical location only, and results may have been strengthened by data from other areas of the body. Dosimeters were also worn external to protective apparel, which results in an overestimation of actual temple dose. It should be noted, though, that this study intended to investigate dose trends with increasing staff height, rather than quantitatively reporting on discrete temple doses.

## Conclusion

It is acknowledged that measuring the effect of one variable during fluoroscopically controlled procedures is problematic. Phantom measurements are useful for investigating parameters in isolation, but findings may be simplistic and may not reflect the complicated clinical reality. Conversely, results that are based on procedural data may have limited transferability due to the many variables that cannot realistically be considered in isolation, given a large number of uncontrollable procedural differences.

This observational study has demonstrated an inverse relationship between combined staff height and occupational temple dose during fluoroscopically guided cardiac examinations but has revealed an important positive relationship between height and temple dose during vascular procedures. Taller nurses performing the role of scrub or scout nurse during vascular angiography may be at greater risk of occupational radiation dose to the head than their shorter counterparts. This is contrary to previous findings in studies with similar procedural parameters investigating non-doctor dose, and the results may reflect the variability of differences in clinical practice. While increased patient and staff dose have been previously noted for vascular procedures, it is unclear the reasons why height in this setting influences dose, and additional research is required to investigate possible explanations. It was also determined that doctor height was correlated with average procedural table height and that vascular access point influences the choice of table elevation.

## Data Availability

The datasets generated during the current study are available from the corresponding author on a reasonable request.
