# Author Correction: Taller staff occupationally exposed to less radiation to the temple in cardiac procedures, but risk higher doses during vascular cases

**DOI:** 10.1038/s41598-020-80349-3

**Published:** 2020-12-30

**Authors:** Kelly S. Wilson‑Stewart, Davide Fontanarosa, Dan Li, Chris C. Drovandi, Rebecca K. Anderson, Jamie V. Trapp

**Affiliations:** 1 School of Chemistry and Physics, Queensland University of Technology, 2 George Street, Brisbane, QLD 4000 Australia; 2 Cardiovascular Suites, Greenslopes Private Hospital, Newdegate Street, Greenslopes, Brisbane, QLD 4120 Australia; 3 School of Clinical Sciences, Queensland University of Technology, 2 George Street, Brisbane, QLD 4000 Australia; 4 Institute of Health and Biomedical Innovation, Queensland University of Technology, Q Block, 60 Musk Avenue, Kelvin Grove, QLD 4059 Australia; 5 School of Mathematical Sciences, Queensland University of Technology, 2 George Street, Brisbane, QLD 4000 Australia; 6 Centre for Data Science, Queensland University of Technology, 2 George Street, Brisbane, QLD 4000 Australia; 7 ARC Centre of Excellence for Mathematical and Statistical Frontiers, The University of Melbourne, Parkville, VIC 3010 Australia

Correction to: *Scientific Reports* 10.1038/s41598-020-73101-4, published online 30 September 2020

The original version of this Article contained errors.

Affiliations 1 and 2 were reversed.

In addition, affiliation 1 was incorrectly given as ‘Queensland University of Technology, 2 George Street, Brisbane, QLD 4000, Australia’.

The correct affiliation is listed below:

School of Chemistry and Physics, Queensland University of Technology, 2 George Street, Brisbane, QLD 4000, Australia

Finally, the original version of this Article omitted an affiliation for Davide Fontanarosa. The correct affiliations for Davide Fontanarosa are listed below:

School of Clinical Sciences, Queensland University of Technology, 2 George Street, Brisbane, QLD 4000, Australia

Institute of Health and Biomedical Innovation, Queensland University of Technology, Q Block, 60 Musk Avenue, Kelvin Grove, QLD 4059, Australia.

These errors have now been corrected in the PDF and HTML versions of the Article.

